# Species-specific microRNA discovery and target prediction in the soybean cyst nematode

**DOI:** 10.1038/s41598-023-44469-w

**Published:** 2023-10-17

**Authors:** Victoria Ajila, Laura Colley, Dave T. Ste-Croix, Nour Nissan, Elroy R. Cober, Benjamin Mimee, Bahram Samanfar, James R. Green

**Affiliations:** 1 Department of Systems and Computer Engineering, Carleton University, Ottawa, K1S 5B6 Canada; 2 Saint-Jean-sur-Richelieu Research and Development Centre, Agriculture and Agri-Food Canada, Saint-Jean-sur-Richelieu, J3B 7B5 Canada; 3 Ottawa Research and Development Centre, Agriculture and Agri-Food Canada, Ottawa, K1A 0C6 Canada; 4 Department of Biology and Ottawa Institute of Systems Biology, Carleton University, Ottawa, K1S 5B6 Canada

**Keywords:** Gene regulation, Computer science, Machine learning

## Abstract

The soybean cyst nematode (SCN) is a devastating pathogen for economic and food security considerations. Although the SCN genome has recently been sequenced, the presence of any miRNA has not been systematically explored and reported. This paper describes the development of a species-specific SCN miRNA discovery pipeline and its application to the SCN genome. Experiments on well-documented model nematodes (*Caenorhabditis elegans* and *Pristionchus pacificus*) are used to tune the pipeline’s hyperparameters and confirm its recall and precision. Application to the SCN genome identifies 3342 high-confidence putative SCN miRNA. Prediction specificity within SCN is confirmed by applying the pipeline to RNA hairpins from known exonic regions of the SCN genome (i.e., sequences known to not be miRNA). Prediction recall is confirmed by building a positive control set of SCN miRNA, based on a limited deep sequencing experiment. Interestingly, a number of novel miRNA are predicted to be encoded within the intronic regions of effector genes, known to be involved in SCN parasitism, suggesting that these miRNA may also be involved in the infection process or virulence. Beyond miRNA discovery, gene targets within SCN are predicted for all high-confidence novel miRNA using a miRNA:mRNA target prediction system. Lastly, cross-kingdom miRNA targeting is investigated, where putative soybean mRNA targets are identified for novel SCN miRNA. All predicted miRNA and gene targets are made available in appendix and through a Borealis DataVerse open repository (https://borealisdata.ca/dataset.xhtml?persistentId=doi:10.5683/SP3/30DEXA).

## Introduction

MicroRNAs, or miRNAs, are a class of short, non-coding RNAs (ribonucleic acids) that work to silence messenger RNA (mRNA). In animals, miRNA synthesis follows a five-step process that includes a pre-miRNA—approximately 70 nt (nucleotides) long with a hairpin structure—as an intermediary step^1,2^. The formation of the mature miRNA is often accompanied by the formation of the ribonucleoprotein miRNA-Induced Silencing Complex (or RISC) that can achieve post-transcriptional gene regulation^3^. MiRNAs can originate from both intragenic and intergenic regions, where the former are mostly derived from intronic regions^4^. Intergenic miRNAs are transcribed and regulated independently from the host genes^4^. Once the mature miRNA-RISC complex is created, it binds with a corresponding target mRNA to regulate its translation or stability. In animals multiplicity exists both ways in this relationship: one mRNA often contains multiple binding areas for miRNA, and correspondingly, one miRNA can affect dozens, if not hundreds, of targets^3^. The miRNA–mRNA binding ultimately means that the miRNA silences its cognate mRNA.

There are many differences between the animal and plant kingdoms concerning biogenesis, miRNA–mRNA binding, and method of miRNA control^5^. A successful plant miRNA–mRNA interaction typically requires a much higher sequence complementarity than animal species in the seed region^5^. Furthermore, homology-based searches of similar miRNA–mRNA relationships in similar species are much more successful in plants than animals^5^. Additionally, the location of miRNA binding site on the mRNA in plants differ from animals^5^. Animal miRNA typically bind in the 3′ untranslated region (UTR) and can exhibit multiplicity, where one mRNA can have many miRNA binding sites and one miRNA can target multiple miRNAs^3,5^. Conversely plant miRNA binds to the target gene’s open reading frame and there is typically only one binding site per mRNA^3–5^.

Moreover, sequence conservation patterns exist up and down-stream of primary miRNA transcripts^6^. These patterns appear to be required for a primary miRNA to be processed into precursor miRNA^6^. However, the conserved sequences appear to differ for nematodes compared to other animals^6^.

### miRNA in plants and pathogens

MiRNAs have been implicated in numerous applications in both animals and plants. Within plants, miRNAs have been linked to biological development and environmental stress adaptations. The overexpression of a miR171 decoy within Arabidopsis, for instance, has been linked to phenotype changes, such as increased rosette leaf area, leaf growth angle, and leaf colour^7^. In nematodes, miRNA have been associated with developmental and metabolic processes and are predicted to regulate the expression of up to 10% of the genes in *Caenorhabditis elegans*^8^. In parasitic nematodes, specific miRNAs were shown to be overexpressed during infection of the host and associated with pathogenicity^9^. The identification of mRNA targets for miRNAs differentially expressed during the transition to infective stages have confirmed that nematodes can use miRNA as a developmental switch that triggers virulence^10^.

Several studies have suggested that a miRNA may not only function in gene regulation within its original cell or species, but also be transmitted in both an intracellular, inter-species, or an inter-kingdom manner^11–15^. This transmission facilitates molecular signalling, communication, and regulation between species and has been observed in several different configurations. Many different pathways for the presence of extracellular miRNA have been hypothesized, including the passive leakage of RNA from broken cells due to cell injury, inflammation, or death; active secretion of the miRNA by way of micro-vesicle-like exosomes; and active secretion using an RNA-binding protein dependant pathway^12,16^. For example, Buck et al demonstrated the exosomal transportation of nematode small RNA into mammalian cells to impact innate cell immunity^17^. Cross-species miRNA:mRNA interactions have been observed between pathogens and hosts, where pathogen sRNA (small RNA) or miRNA target host genes or host RNA target pathogen genes^15,18–26^. A 2016 study demonstrated that cotton plants responded to infection with Verticillium dahliae, a fungal plant pathogen that causes wilt diseases in many crops, with increased production of miR166 and miR159 and exportation of these miRNAs to the fungal hyphae to function in specific silencing^27^. These miRNAs targeted the V. dahliae genes Clp-1 and HiC-15, respectively, both of which are vital to the fungus’ ability to infect its host^27^. Conversely, a 2018 study identified several *Arabidopsis thaliana* mRNAs targeted by miRNAs from the parasitic plant Cuscuta campestris^28^. The targeted mRNAs included BIK1, which encodes a kinase required for signalling, and HSFB4, which encodes a transcriptional repressor important for the development of ground-tissue stem cells in roots^28^. Additionally, a recent study demonstrated the suppression of the pathogen Botrytis cinerea in vitro by novel tomato miRNA^29^.

The presence of a pathogen, virus, or parasite can initiate differential expression of miRNA within a host organism. This has been observed among plants infected with nematodes^30,31^, including among soybean plants infected with soybean cyst nematodes (Heterodera glycines, or SCN). A 2019 study identified 40 soybean miRNAs—14 previously known, and 26 novel ones—that may be implicated in the soybean response to SCN infection^32^. Similarly, Li et al. identified a total of 101 soybean miRNAs that were significantly differentially expressed in response to SCN infection^33^. These miRNAs were from 40 families and all but 6 were down-regulated^33^. Tian et al. identified 60 miRNAs belonging to 25 families related to SCN infection^34^. Rambani et al. demonstrated the differential methylation of miRNA genes within the soybean genome in response to SCN infection resulting in the overexpression of 4 miRNA^35^. The over- and under-expression of exocyst genes in soybean have been linked to the suppression and facilitation of SCN parasitism^36^. The functions of the genes targeted by these miRNAs are often not fully understood; in many cases, however, they are hypothesized to be related to plant defence^32,34^.

### Computational discovery of miRNA

The identification of miRNA is an important area of research, considering the significant role miRNA play in biological processes. The methods used to identify and discover miRNA are highly interdisciplinary^37^. MiRNA can be identified experimentally through costly and time consuming wet-lab verification techniques or computationally using a variety of techniques^37–39^. For example, to perform wet lab verification of potential miRNA in SCN requires the growth of soybean plants for a full season. The nematodes need to be carefully hand-picked from the soybean roots to avoid soybean contamination before the isolation and sequencing experimental process begins. Computational techniques can be separated into two categories, homology-based and machine learning-based^40^. Homology-based techniques use sequence similarity from previously identified miRNA to predict new miRNA^40^. These predictors can confidently identify homologue miRNA across different species; however, they cannot predict novel miRNA that are unique to the target species^40^. Methods that leverage supervised machine learning (ML) can be further separated into two techniques, sequence-based (de novo) techniques or expression-based techniques, where the latter uses next-generation sequencing (NGS) to quantify expression^37–40^. De novo prediction techniques classify miRNA based on features describing its sequence and secondary structure^40^. De novo techniques must examine all miRNA-like hairpin structures in the entire genome, which leads to a significant class imbalance due to a large number of candidate miRNA in the genome^38,41^. Conversely, NGS-based techniques need only consider expressed regions rather than the whole genome^41^. NGS data describe both the sequence and quantity of the expressed RNA in a sample^41^, which may arise from mRNA degradation products, microRNA, or other non-coding RNA (ncRNA)^38^. With the increasing availability of NGS, NGS-based miRNA discovery techniques have become increasingly popular; however, it is worth noting that these techniques can be biased to miRNA with high expression levels^40^. Unlike de novo prediction techniques, NGS-based techniques do require transcriptomic data, which is not available for a large number of organisms of interest^39,41^. Also, sequencing only captures miRNA’s expressed under the specific conditions used (e.g. developmental stage, host suitability, temperature, food availability, etc.) making it challenging to capture the full diversity of miRNA expressed within an organism.

The training sets of significant miRNA discovery studies are typically formed by retrieving positive miRNA data from the miRBase database and negative data from ncRNA and protein-coding exonic sequences that form a similar structure to pre-miRNA^40^. There are only a relatively small number of known miRNA; the miRBase database, for example, only contains miRNA for less than 300 species and 30% of those species have 15 or fewer known miRNA^38^.

Our group has recently developed the Species-specific MIRna Predictors (SMIRP) technique to dynamically create sequence-based species-specific training data for the generation of miRNA classifiers^40^. SMIRP was shown to be particularly effective when developing miRNA predictors for under-studied species since it creates a large, highly conserved, and non-redundant miRNA training dataset, while giving preference to exemplars from species most closely related to the target species. MiRNA-like hairpins from closely related species are used to develop negative training data^40^. SMIRP provided an increase in miRNA predictor performance for four distinct species in comparison to other dataset generation methods^40^. Performance increases were shown to be conserved across different classification models^40^.

### Computational miRNA target prediction

In addition to miRNA discovery, it is also important to identify the corresponding mRNA targets. Usually, mRNA targets are painstakingly identified using experimental techniques, like biochemical assays^42^. Experimentally validated miRNA–mRNA pairs can be found in repositories such as miRWalk^43^, miRecords^44^, TarBase^45^, miRTarBase^46^, and starBase^47^. However, in many cases the miRNA–mRNA pairs present in these databases have been validated using reporter assays.

Given the benefits of computational miRNA target prediction tools, a number of ab initio predictors have been developed based on these data repositories. MiRNA target prediction rules were defined based on features such as the sequence complementarity of different locations of the seed and target site, the thermodynamic stability of the duplex, the accessibility of the target site, AU content, folding energy, conservation, a perfect pairing of the miRNA 5′ end, and low GC-content in the target site^48,49^. MiRanda^50^ is an ab initio method that uses an estimated complementarity score, conservation, and free energy values to predict target sites^48,49,51^. TargetScan^52^ is an ab initio method that looks for perfect seed matches to comprise a candidate target list then uses site-type, local AU enrichment, and other features to calculate a target score^49,52^. MicroTar^52^ and FindTar^53^ are ab initio methods that allow for G:U wobbles by considering different complementarities in the seed in their prediction methodologies^49^. psRNATarget^54^ is a plant-specific ab initio method that makes use of a modified Smith–Watermen algorithm and the RNAup algorithm^55^ to discover high-confidence miRNA targets^56^. Other plant-specific ab initio algorithms like Targetfinder^57^, TAPIR^58^ and Target-align^59^ use the Smith–Watermen algorithm or the FASTA program along with scoring methods to discover high-confidence miRNA:mRNA interactions^56^. miRTour^60^ and Target_Prediction^61^ discover high-confidence miRNA:mRNA interactions based on energy minimizations such as the calculation of minimum free energy of a miRNA:mRNA pair^56^. Targetfinder combined with psRNATarget has been shown to show favourable results^56^.

Several ML-based miRNA target prediction methods have also been developed where feature patterns are derived from experimentally verified data to post-filter predictions from ab initio algorithms^49^. The RFMIrTarget method applies a random forest classifier based on 17 features extracted from a miRanda prediction set^49,62^. MultiMiTar applies a support vector machine on 90 features of the miRNA:mRNA pair selected by a multi-object metaheuristic technique^49,63^. TarPMiR is a random-forest-based approach that integrates six conventional features with seven new features to predict miRNA target sites^48^. TarPMir was shown to outperform two TargetScan versions and one miRanda version across human and mouse datasets, particularly for non-seed-matching binding sites^48^. NBmiRTar is a hybrid technique that first applies the miRanda algorithm then applies a Naïve Bayes 57 feature classifier to filter the output^49,64^. Several repositories for predicted interactions exist, including EIMMo^65^, DIANA-microT^66^, Microrna.org^67^, TargetScan^68^, MirDB^69^, miRWalk-predictive^70^, and TargetSpy^71^.

Although most ML methods have been trained and validated on animal miRNA:mRNA interactions, many can be retrained using plant interaction data to improve miRNA target prediction in plant species. p-TAREF is a plant-specific ML algorithm that applies Support Vector Regression to position-specific dinucleotide density variation information from the target sites^72^.

### miRNA discovery and target prediction in SCN

Canada and the United States are both major producers of soybean (Glycine max), with more than 21.3 billion bushels produced between 2015 and 2019 which were valued at over 191 billion USD^73^. Soybean diseases and pathogens can reduce the quality of grains as well as reduce yield^73^. Between the years 2015 and 2019, in Ontario and the United States, the SCN was the most destructive pathogen and caused twice the loss of any other diseases^73^. This nematode is an obligate endoparasite of soybean roots, where it forms a giant multinucleated feeding structure called the syncytium^74^. While still not fully understood, this complex interaction between nematode and plant, leading to the formation of the syncytium, is thought to arise from SCN secreted molecules called effectors^75^. Yet, there is still limited information on the cellular processes responsible for the regulation and expression of these effectors but also on how these effectors interact with the host^75^. As such, gathering more insight into the interaction between soybean and SCN is essential as it could lead to a more effective and efficient control modality. Management of SCN infections in soybean crops has typically involved the use of crop rotations and nematode-resistant crop varieties^76^. Unfortunately, more than 95% of the resistant cultivars are derived from a single source: PI 88788, which has led to the selection of virulent populations^74^. Therefore, the current tools to control SCN have limited effectiveness and long-term sustainability^75^. Newer strategies have been developed that explore and exploit natural plant stress responses. Plants alter their gene expression before, during and after transcription to reduce damage caused by a stressor^33^. Small RNAs like miRNA are important participants in the gene regulation process^33,77^. Beyond intra-species gene regulation, a recent thesis has suggested that SCN can secrete a small set of miRNA that targets the host mRNA during parasitism^78^. The thesis reports on the discovery of 21 potential miRNA through the application of expression-based miRNA discovery algorithms to a currently unpublished set of small RNA sequencing data^78^. The thesis also predicts 15 SCN-soybean miRNA:mRNA interactions with high-confidence^78^.

In this paper, we present a species-specific ML pipeline to identify novel miRNA in SCN, an important pathogen with few documented miRNAs. The pipeline discovered 3342 high-confidence miRNA within SCN. We go on to predict the inter-species (SCN and soybean) and SCN intra-species mRNA targets for the identified putative miRNA. The novel intra- and inter-species miRNA discovery and target prediction methodology developed here is also applicable to other plant pathogens.

## Methods

This study has two principle phases: miRNA discovery in SCN, and mRNA target prediction for the putative miRNA in both SCN (intra-species) and soybean (inter-species).

### miRNA discovery

To develop a species-specific miRNA discovery pipeline for SCN, datasets and predictors were developed for three nematodes: *Caenorhabditis elegans* (CE), *Pristionchus pacificus* (PP), and SCN. The first two species represent model species for which substantial ground truth data were available for validating our pipeline.

#### Candidate pre-miRNA Set

MiRNA discovery involves the application of a ML model to a set of candidate putative pre-miRNA. The candidate pre-miRNA dataset was determined by retrieving the organism’s genome assembly and extracting 500 nt long sequences with a stride of 250 nt from the assembly. The reverse complement sequences were ascertained as well. RNALfold^55^ was applied to the sequences to extract sub-sequences with secondary structures. The sub-sequences were filtered such that the sub-sequences with a minimum free energy of less than − 25 kcal/mol, a perfect stem (no structural bulges) with a length greater or equal to 25 and a sequence length of less than 150 remained; this formed a set of candidate pre-miRNA. To remove duplicate hairpin sequences, the CD-HIT program^79^ was used to cluster the sequences with a conservative sequence identity threshold of 90%. The sequence that was the most representative of each cluster as determined by CD-HIT was chosen for the final SCN candidate pre-miRNA dataset. All sequences derived from exonic regions were excluded from the candidate set. Then a BLAST^80^ was used to identify any duplications or near duplicated hairpins remaining in the data set at a minimum e-value of 10^-10^; no such sequences were identified. The HeteroMirPred program^81^ was then applied to the candidate pre-miRNA to generate the sequence-based features.

#### Training set development

To train miRNA discovery algorithms, a set of positive and negative training examples were required. SMIRP—a method of creating species-specific sequence-based training data—was used to define positive and negative training sets. The algorithm aggregates known miRNA data from multiple species, giving preference to highly conserved miRNA and exemplars from species phylogenetically close to the target organism, resulting in a dataset suitable for training ML approaches to miRNA discovery^40^ . Known pre-miRNA from many organisms are first clustered by CD-HIT^79^ using an 80% sequence identity threshold. The representative sequence from each cluster that is phylogenetically closest to the target species was chosen to form the positive training set^40^ . Similar to the candidate set generation, a sliding window of length 500 nt at a stride of 250 nt was used to extract sub sequences from each organism represented in the positive training set. RNALfold^55^ was used to extract hairpins from the sub sequences and the hairpins were filtered using the same criteria as the candidate set. BLAST^80^ was used to find the matching hairpins (hairpin with the smallest e-value) for each positive miRNA. The process was performed so that there were no procedural differences between the ascertainment of positive, negative and candidate hairpins.

The negative sequence-based training set was created from a nematode genome and comprised RNA known to not form miRNA, like coding RNA and non-coding RNA with functions other than miRNA (e.g., snoRNA, siRNA, tRNA, etc.)^40^ . Sequences that could form secondary structures were extracted from the coding RNA and other ncRNA using the RNALfold program^55^ . The sequences that did not have a minimum free energy of less than − 15 kcal/mol or a stem length greater or equal to 18 were discarded, creating a set of hairpin-like sequences. Similar to the positive training data, negative hairpin sequences were clustered using the CD-HIT at a sequence identity of 90% to remove duplicate sequences^79^ and the representative sequences from each cluster formed the negative set. The HeteroMirPred program^81^ was used to extract a total of 215 sequence-based features from the positive and negative training sets^40^.

#### SCN positive control validation set

To develop a list of positive SCN miRNA for validation of the classification pipeline, putative miRNA discovered in SCN using a limited read depth NGS experiment were retrieved (NCBI BioProject PRJNA951618). BLAST^80^ was used to map the positive miRNA with the corresponding sequence in the SCN candidate set. These corresponding sequences comprised the SCN positive control validation set. Negative SCN validation data were defined as those pre-miRNA-like hairpins extracted from the SCN genome that mapped to exonic regions of the genome.

#### Classification pipeline

The miRNA discovery classifiers in this study took the form of a random forest classifier of 500 trees built using the ensemble package in the SKLearn library^82^. This design choice followed the results in^40^. In the first experiment (PP-holdout), we simulated an environment where the genome of a model species (*P. pacificus*) within the nematode phylum was unannotated. SMIRP was used to create a *P. pacificus*-specific training set using a modified miRNA database that excluded *P. pacificus* pre-miRNA. Positive examples were extracted from the modified database and negative examples were extracted from only *C. elegans* protein-coding RNA, tRNA, and rRNA. The classifier was trained on these examples and applied to a test set containing all known *P. pacificus* miRNA in miRBase and negative examples extracted from *P. pacificus* coding RNA, tRNA, and rRNA. A second experiment (CE-holdout) was performed with a similar methodology as stated for Experiment 1 except with *C. elegans* and *P. pacificus* in reverse positions.

A third experiment was performed utilizing the entire miRBase database. The positive dataset was created using SMIRP and negative examples were extracted from *C. elegans* and *P. pacificus* protein-coding regions and other ncRNA. The resultant nematode classifier (PP + CE) was applied to candidate pre-miRNA dataset extracted from the SCN genome, including those that comprise the positive and negative validation sets described above.

#### Class imbalance estimation

The training sets described above exhibit a relatively small class imbalance, while in practice the ratio of true pre-miRNA to hairpin regions with similar length and MFE in SCN would be far more extreme. To account for the extreme class imbalance expected when the predictors are applied to entire genomes, the class imbalance was estimated in *C. elegans* and *P. pacificus*. Similar to the candidate set generation process, a sliding window of length 500 nt and stride 250 nt was applied to the genome of both *C. elegans* and *P. pacificus*. RNALfold was applied to extract hairpins from the subsequences. The same filter was applied to remove any sequences with a MFE of greater than − 15 kcal/mol or a stem length less than 18. All sequences derived from exonic regions were excluded. BLAST^80^ was applied to hairpins and the known miRNA in each organism to locate the hairpins containing a true/known pre-miRNA. The number of hairpins containing a known pre-miRNA was compared to the number of pseudo-miRNA hairpin regions, thereby estimating class imbalance.

### miRNA target prediction

Mirdup, a computational predictor for the mature miRNA from a pre-miRNA sequence was used to extract the mature miRNA from the high-confidence pre-miRNA candidate set^83^. A modified TarPMir miRNA target predictor was applied to predict interactions between the high-confidence mature SCN miRNA and SCN mRNA. The TarPMir miRNA:mRNA target prediction method was originally trained on human CLASH experimental data. The dataset contained 18,514 positive examples and 18,514 negative examples of interactions involving 399 miRNAs^48^ . The training set of the original TarPMir target predictor was augmented with 173 intraspecies *C. elegans* miRNA–mRNA targets^68,84^. Five-fold cross validation using the augmented training set demonstrated that the addition of *C. elegans* data significantly improved precision and accuracy while maintaining a similar recall to the original TarPMir classifier. The newly trained model (CE-TarPMir) was applied to the SCN miRNA and SCN mRNA. Similarly, a classifier utilizing the same prediction architecture as TarPMir was trained on data derived from TarDB^85^, a database of intraspecies plant miRNA:mRNA interactions. The Plant TarPMir classifier (P-TarPMir)^86^ was applied to predict interactions between the high-confidence SCN mature miRNA and soybean mRNA. CE-TarPMir was applied to the high-confidence candidate mature SCN miRNA and all available SCN mRNA. P-TarPMir was applied to the high-confidence mature SCN miRNA and 216 soybean mRNA which could be involved in the defence of pathogens. The list of soybean genes were determined based on literature curation as broken down in Supplementary File S4 as well as from Soybase’s GWAS QTL page under SCN 1–SCN 6^87^. Gene names as in version Wm82.a2.v1. The results were filtered such that only the highest confidence binding site interaction for a miRNA:mRNA pair remained. Additionally, in the case of the SCN intra-species target predictions, only the interactions that occurred in the 3′ untranslated region (UTR) of the SCN mRNA were retained.

A qualitative reciprocal perspective approach was utilized to define high-confidence lists of intra-species and inter-species targets. Reciprocal perspective has been used by RPmirDIP to significantly improve miRNA target prediction performance by leveraging the two complementary views of a miRNA–gene pair to develop confidence thresholds^88^. However, RPmirDIP trains a predictor on experimentally validated targets of an organism to determine thresholds that are not available for SCN^88^. Instead, a qualitative approach was utilized to reduce the set of interactions predicted by TarPMir and P-TarPMir. For thresholds n and m, we retain only miRNA:mRNA pairs where the miRNA is among the top-n predicted partners for the mRNA and the mRNA is among the top-m predicted partners for the miRNA. By varying n and m in the range [1, 4, 8, 10, 25, 50, 100], progressively more permissive candidate high-confidence miRNA:mRNA interaction sets are formed.

## Results and discussion

### miRNA discovery

The SMIRP algorithm was used to develop training sets for three miRNA discovery experiments. The first experiment simulated the case where the miRNA and coding regions of the nematode *P. pacificus* are unknown. The second experiment simulated the case where the miRNA and coding regions of *C. elegans* are unknown. The third experiment simulated the case where the coding regions of SCN are unknown. Table 1 displays the size of the training and test/validation sets for each experiment.

**Table 1:** Size of training set prepared for the three miRNA discovery experiments.

Experiment	Test species	Size of positive training set	Size of negative training set	Size of positive test set	Size of negative test set
1 (PP hold-out)	*P. pacificus*	934	1575	338	1000
2 (CE hold-out)	*C. elegans*	932	2156	242	1000
3 (PP + CE)	SCN	947	1682	66	66

The set of candidate pre-miRNA retrieved from the SCN genome comprised over 225 thousand sequences after clustering from over 1 million extracted hairpin sequences. Sequences from clusters containing exonic regions were removed, reducing the candidate set to 113,985 hairpin sequences.

#### Validation of SMIRP for nematodes

**Figure 1:**
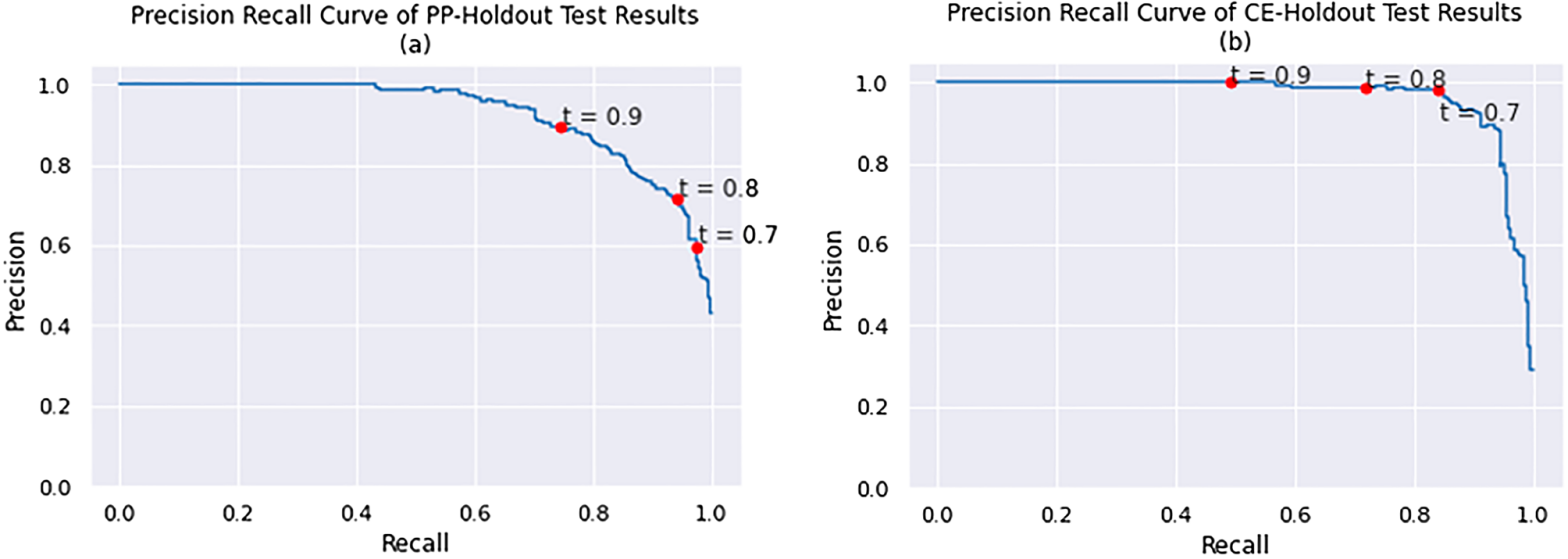
The precision–recall curves of (**a**) PP-holdout classifier and (**b**) CE-holdout classifier applied to their test datasets.

**Figure 2:**
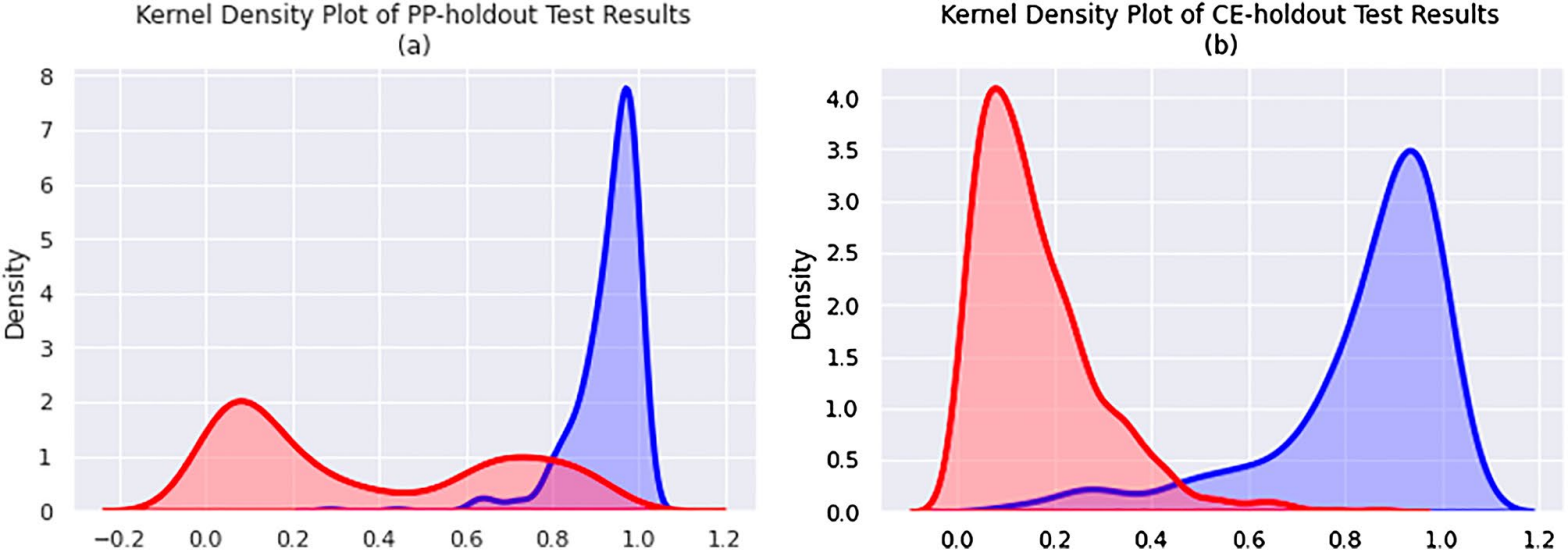
The kernel density curves of prediction scores (negative = red, positive = blue) for the (**a**) PP-holdout classifier and (**b**) CE-holdout classifier applied to their respective test datasets.

SMIRP, a species-specific miRNA training set generation framework, has been shown to improve miRNA classification performance^40^ . The SMIRP algorithm allows for the use of positive and negative miRNA from a multitude of species while preferring miRNA with greater phylogenetic similarity to the target species^40^ . Since SMIRP had not previously been validated for use in nematodes, two validation experiments were conducted with well-annotated species: *C. elegans* (CE) and *P. pacificus* (PP). As described above, the PP-holdout experiment involved the construction of a PP-specific classifier using SMIRP, when all PP known miRNA were excluded from the training dataset. Negative training data were taken from CE. The CE-holdout experiment was identical, with the PP and CE roles reversed. Figures 1 and 2 show the results from the PP- and CE-holdout experiments.

Excellent separation of the positive and negative test sequences in Fig. 2 led to the very strong precision-recall curves in Fig. 1. Table 2 summarizes the performance of the PP-holdout classifier and CE-holdout classifier on their test set at two thresholds.

Peace et al. demonstrated the utility of the SMIRP framework from a number of species across plants, animals, and viruses however these experiments are the first application of SMIRP to nematodes specifically^40^ . To conclusively validate the benefit of SMIRP on nematodes, these results were compared to an otherwise identical miRNA discovery model trained on human pre-miRNA only. For both nematode species, SMIRP showed a small but consistent improvement across all performance metrics. The results can be seen in Supplementary Figures S1, S2 and Supplementary Table S3. The increase of performance from the human classifier to SMIRP shows the benefit of creating species specific classifiers, trained on data from closely related phylogenetic species.

**Table 2:** The average area under precision recall curve (AUPRC), precision, recall and accuracy of PP and CE-holdout classifiers at on test sets at three thresholds.

Exp.	AUPRC	Threshold	Recall	Precision	Accuracy
PP-holdout	0.930	0.7	0.974	0.597	0.829
		0.8	0.942	0.716	0.890
		0.9	0.743	0.895	0.912
CE-holdout	0.966	0.7	0.839	0.981	0.965
		0.8	0.719	0.989	0.944
		0.9	0.492	1.000	0.901

Both the PP- and CE-holdout experiments resulted in precision and accuracy values near or above 0.9 at the threshold of 0.9. Examining Figs. 1 and 2, a threshold of 0.8 can be applied to discriminate the positive test examples from the negative test examples. Notably PP- and CE-holdout experiments, for a recall of at least 0.5, precision reached 0.98 and 1.0, respectively. Such strong miRNA discovery performance is partially explained by the phylogenetic similarity between the species included in the training and test data (e.g., *C. elegans* in the training data and *P. pacificus* in the test data, or vice-versa). It is noted that SCN is significantly more evolutionarily distant from the training species than in either the CE- or PP-holdout experiments, as reported here^89^ . Peace et al. have previously shown that such increased evolutionary distance between train and test species leads to reduced miRNA predictive performance^40^ . Lastly, the training and testing datasets both exhibit low levels of class imbalance, which also simplifies the prediction task.

#### SCN validation set performance

**Figure 3:**
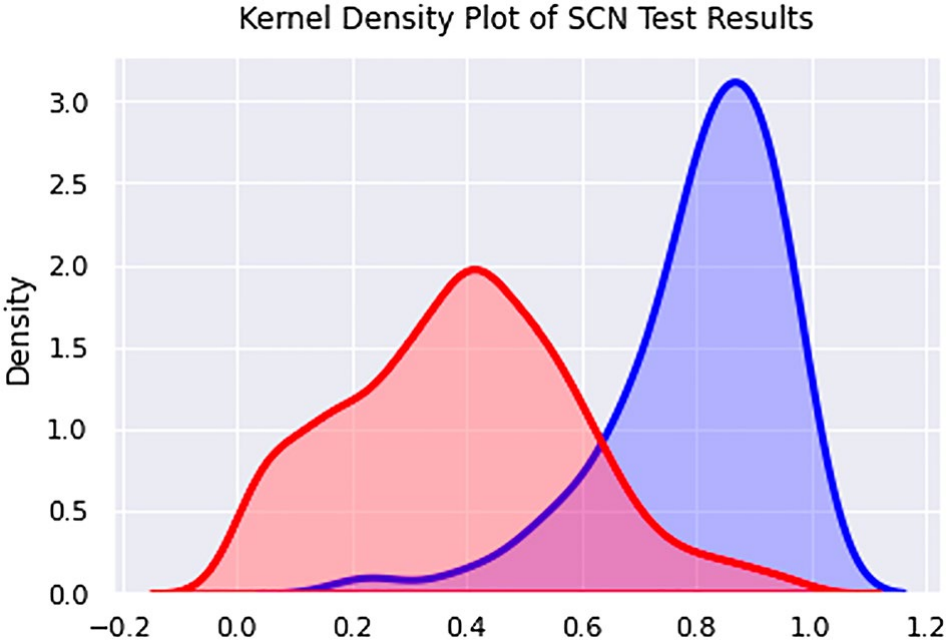
The kernel density curve of PP + CE classifier applied to the positive and negative SCN validation sets.

**Figure 4:**
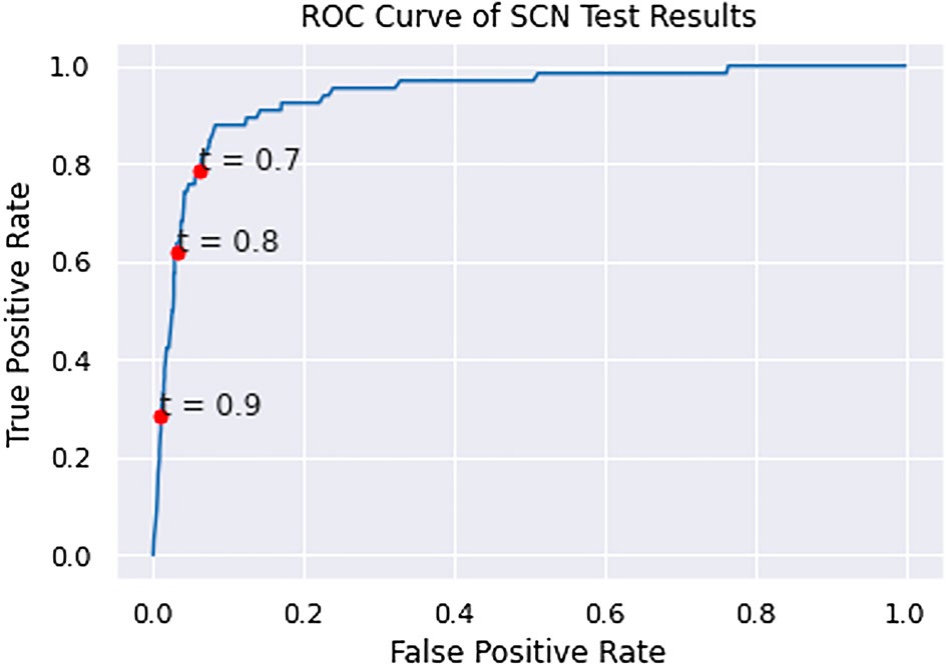
The ROC curve of PP + CE classifier applied to the positive and negative SCN validation sets.

The positive control validation set was comprised of 66 candidate hairpins that match putative miRNA found in a shallow SCN NGS experimentation (see Table S5 in Supplementary Materials). The third and final SCN miRNA predictor was trained without excluding any species in the positive training set and included hairpins extracted from protein-coding RNA, tRNA, and rRNA from *C. elegans* and *P. pacificus* in the negative training set. This resulted in a generalized nematode classifier. The specificity of the PP +CE classifier was measured using pseudo-miRNA from exonic regions of SCN. The performance of the PP + CE classifier on the positive and negative SCN validation sets is shown in Figs. 3 and 4.

Figure 3 illustrates a greater overlap in positive and negative prediction scores for SCN, compared to the CE- or PP-holdout experiments. This leads to substantially reduced performance in the ROC curve illustrated in Fig. 4. Performance at decision thresholds of 0.8 and 0.9 is summarized in Table 3.

**Table 3:** The performance of the PP + CE classifier on the SCN test set at three thresholds.

Threshold	Recall	Precision	Accuracy
0.7	0.788	0.444	0.929
0.8	0.621	0.562	0.949
0.9	0.288	0.633	0.948

Experiment 3 demonstrated that a generalized nematode classifier trained on a SCN SMIRP dataset can recognize negative examples in SCN with high specificity. The PP + CE classifier was also able to recover over half of the positive control validation set at a confidence threshold of 0.8. Approximately 88% of the positive validation set is above the 90th percentile among the prediction confidences of the candidate set. Additionally, approximately 98% of the prediction confidences of the negative validation set are below the 90th percentile of the prediction confidences of the candidate set, implying that 88% of the positive validation set scored higher than 98% of the negative validation set, indicating strong separation between positive and negative sequences.

A significant drop in performance can be seen between PP- and CE- holdout experiments and Experiment 3. This can be attributed to the phylogenetic distance between *C. elegans* and *P. pacificus* and *C. elegans* and SCN. The class imbalance in the SCN test set ( 1:150) is also higher than those of the CE- ( 1:4) and PP-holdout ( 1:3) tests. Note that a fourth classifier was trained on data including SCN exonic regions in the negative training dataset. The classifier did not result in improved performance on the validation set. Therefore, the classifier from Experiment 3 is used to make predictions on the candidate pre-miRNA set.

#### Accounting for class imbalance: prevalence-corrected performance

**Figure 5:**
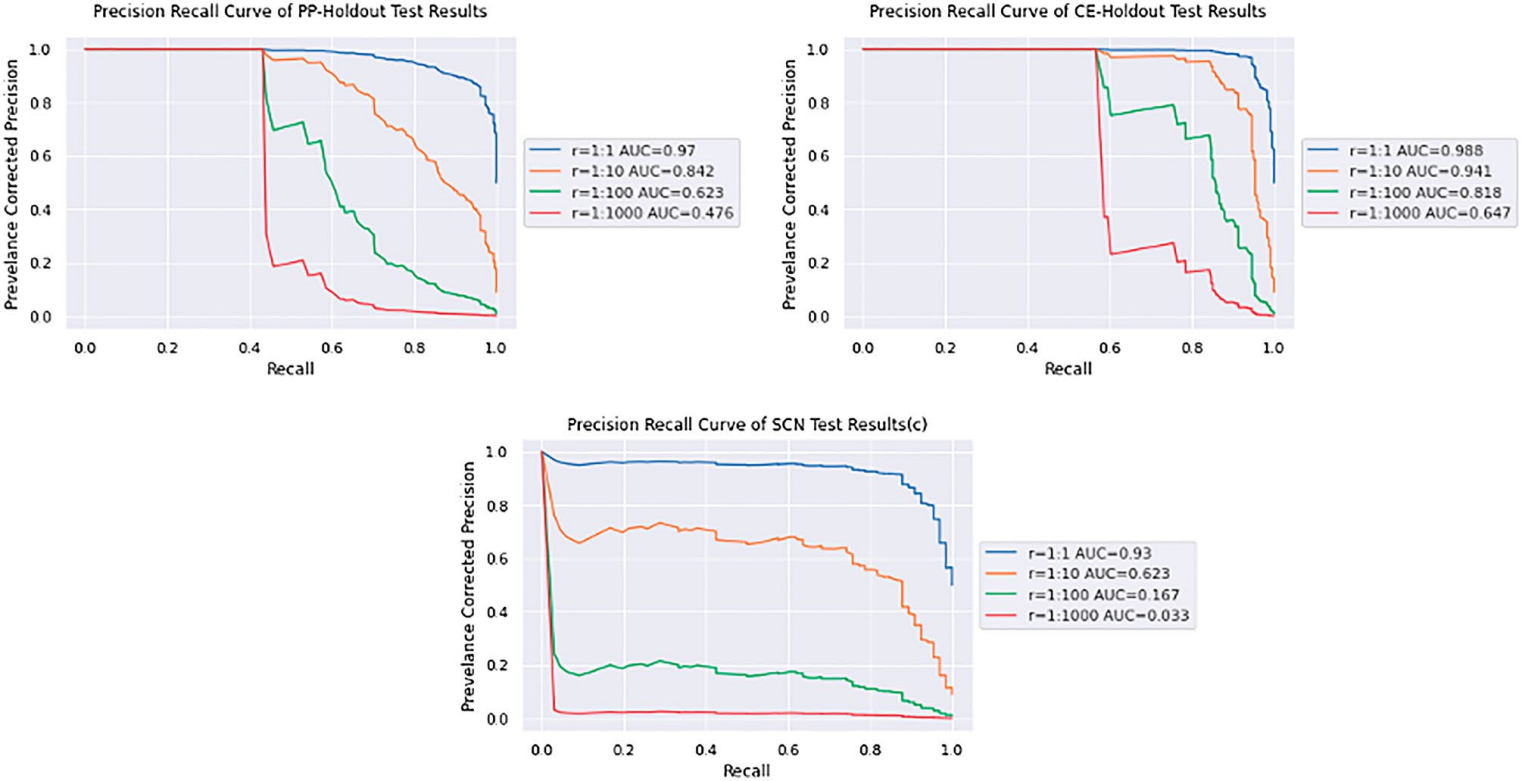
The prevalence-corrected precision recall curves for PP-holdout, CE-holdout, and PP + CE classifier.

The class imbalance of hairpins containing pre-miRNA to other hairpins in *C. elegans* and *P. pacificus* was estimated to be 1:1000. That is, there are approximately 1000 pseudo-miRNA hairpin regions for each true miRNA. Given such a large class imbalance, the precision and AUPRC were recalculated using prevalence-corrected precision for all three experiments. Figure 5 and Table 4 displays the corrected results. A class imbalance of 1:1000 was used for all three cases to more fairly compare them.

**Table 4:** The prevalence-corrected AUPRC, recall and precision at 1:1000 for experiments PP-holdout, CE-holdout, and PP +CE classifier.

Exp.	AUPRC	Threshold	Recall	Precision
PP	0.476	0.7	0.974	0.004
		0.8	0.942	0.007
		0.9	0.743	0.024
CE	0.647	0.7	0.839	0.173
		0.8	0.719	0.264
		0.9	0.492	1.000
SCN	0.033	0.7	0.839	0.012
		0.8	0.719	0.020
		0.9	0.492	0.027

**Figure 6:**
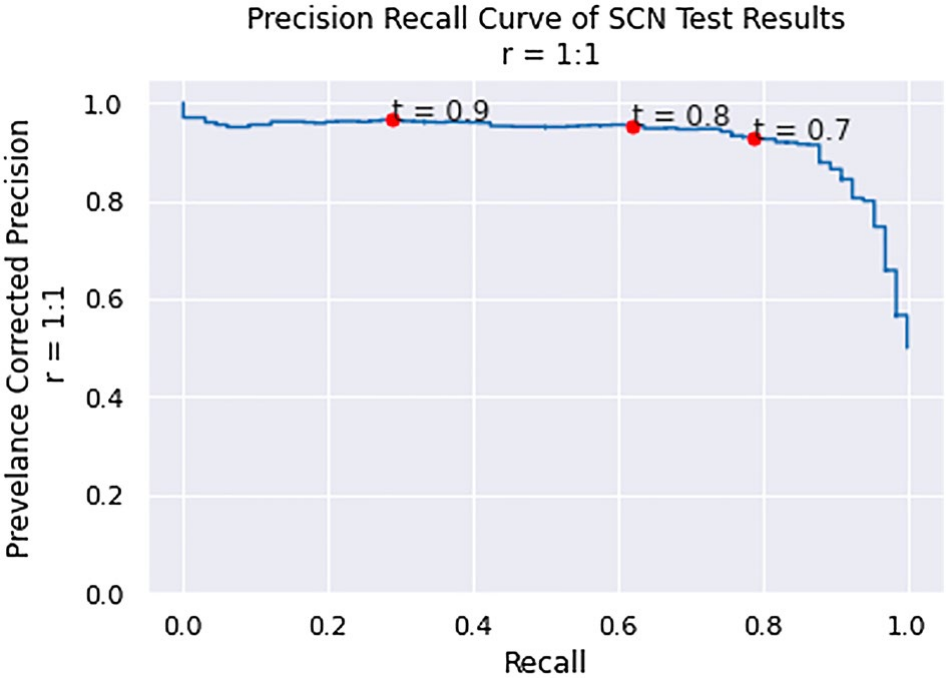
Precision recall curve of PP + CE classifier corrected to a “balanced” test set.

Figure 6 and Table 4 demonstrate substantial reduction in performance when a realistic class imbalance is used. Recall is relatively stable; however, as the number of pseudo-miRNA sequences increases, the number of false positive predictions increases rapidly, negatively affecting the precision. It is uncommon for these results to be reported; many methods like^90–94^ report performance estimates derived from “balanced” test sets, or test sets with a relatively small class imbalance (< 1:20). To illustrate the optimistic bias resulting from the naïve assumption inherent in a “balanced” test set, prevalence corrected precision is used to estimate the performance of the PP + CE classifier when applied to a “balanced” SCN test set, as shown in Fig. 6 and Table 5.

**Table 5:** PP + CE classifier performance corrected to a balanced test set.

AUPRC	Threshold	Recall	Precision
0.930	0.7	0.788	0.927
	0.8	0.621	0.953
	0.9	0.288	0.965

#### SCN candidate set predictions

Table 6 display the distribution of the predicted miRNA confidences for each SCN candidate pre-miRNA.

**Table 6:** Distribution of PP + CE classifier prediction scores on the SCN pre-miRNA candidate set.

Prediction score range	Count
0–0.10	6513
0.10–0.20	7854
0.20–0.30	12,379
0.30–0.40	17,520
0.40–0.50	22,173
0.50–0.60	22,033
0.60–0.70	14,984
0.70–0.80	7247
0.80–0.90	2755
0.90–1.00	587

The application of the PP + CE classifier the SCN pre-miRNA candidate set resulted in 587 sequences (0.5% of the candidate set) predicted to be miRNA with score $\ge$ 0.9, and 3342 sequences (3% of the candidate set) predicted to be miRNA with score $\ge$ 0.8. Here, prediction score is a proxy for prediction confidence. Ultimately, a score threshold of 0.8 was applied since this represented an uncorrected precision of approximately 62%. The final ranked list of these high-confidence SCN pre-miRNA contained 3342 hairpins comprising those that scored greater or equal to 0.8. All predicted high-confidence miRNA have been made available in Table S6 in supplementary materials and through a Dataverse open repository: https://borealisdata.ca/dataset.xhtml?persistentId=doi:10.5683/SP3/30DEXA. Additionally a BLAST^80^ experiment was performed to establish the known mature miRNA in miRBase sharing the greatest homology with each of the predicted mature miRNA listed in Table S6. The names of these homologous mature miRNA are also available in Table S6.

A recent study produced a list of 21 SCN sequences predicted to be miRNA by miRDeep2 from a publicly unavailable small RNA sequencing dataset^78^ . Of the 21 sequences found in^78^ , all 21 sequences were found in our pre-miRNA candidate set and 9 of those sequences appear in our final list of high-confidence SCN pre-miRNA. These results are not unexpected as the PP + CE classifier has a recall of less than 2/3 at a conservative threshold of 0.8 Moreover, given that the methodology described in Barnes et al.^78^ does not have a perfect precision a FPR of greater than zero can be expected for the SCN miRNA predictions described.

Of the 3342 high-confidence SCN miRNA discovered, 1259 sequences were found in intronic regions, 123 of which are among the SCN genes thought to play a role in virulence. Among these, five are of particular interest because they were located in genes (Hetgly05026, Hetgly08659, Hetgly14753, Hetgly16169, Hetgly19158) confirmed as either effector genes or genes shown to be differentially expressed in resistant and susceptible soybean cultivars^95,96^. If the transcription of these intronic miRNA precursors is co-regulated with the gene hosting them, their expression would synchronize with key moments dictating the outcome of pathogenicity or virulence. We can therefore hypothesize that these miRNAs could either influence gene expression in the host or switch the nematode’s own expression profile to a virulent mode.

In order to further establish the plausibility of the putative miRNA identified by our proposed miRNA discovery pipeline in SCN, each of the eight criteria capturing the unique structural features of miRNA were examined, as defined by the miRGeneDB project (see https://www.mirgenedb.org/information). Criteria 2 and 4 require expression data and could not be assessed directly. Criterion 8 relates to patterns of sequence conservation in the primary miRNA transcript; this cannot be assessed given that our pipeline begins at the extraction of candidate precursor miRNA. However, the remaining criteria were analyzed. Criterion 1 states that two 20–26 nt long reads are expressed from each of the two arms derived from a hairpin precursor. Criterion 3 states that the hairpin precursor shows imperfect complementarity, and base pairs in at least 16 of the   22 nucleotides. Our pipeline does not include expression analysis however, we can confirm that all putative miRNA identified by the pipeline have a stem length between 20 and 25 nt without bulges. This indicates that the length of expressed mature and passenger strands should fall within the specified range with sufficient binding complementarity. Criterion 5 states that the length of the loop should be between 8 and 40 nucleotides. The resulting high-confidence list from our pipeline had loop lengths ranging between 3 and 40 nucleotides and therefore meet the criterion. Criterion 6 states that the mature microRNA sequence usually starts with A or U, and is often mismatched with the complementary arm. Of the 3342 high-confidence predicted miRNA 3046 have a mature sequence starting with A or U. Criterion 7 states that nucleotide positions 2–8 and 13–16 of the mature sequence are strongly conserved through evolution. To estimate sequence conservation, we used the most similar known miRNA from the BLAST experiment (see Table S6) to determine if these regions were conserved in the candidate pairings. Relative to their most similar homologs, of the 3342 high-confidence miRNA, 879 candidates have sequence conservation for nucleotides 2–8, 2598 exhibit sequence conservation for nucleotides 13–16, and 355 are conserved over both ranges.

### miRNA target prediction

Intra-species and cross-kingdom inter-species miRNA target prediction was performed for each of the high-confidence SCN miRNA discovered during SCN miRNA discovery. TarPMir, an algorithm originally trained on human and mouse miRNA:mRNA interactions and augmented with *C. elegans* targeting data was used to predict the relationships between SCN miRNA and SCN mRNA. P-TarPMir, an algorithm trained on the plant interaction database TarDB, was used to predict interactions between SCN miRNA and soybean mRNA.

#### SCN intra-species miRNA target prediction

**Figure 7:**
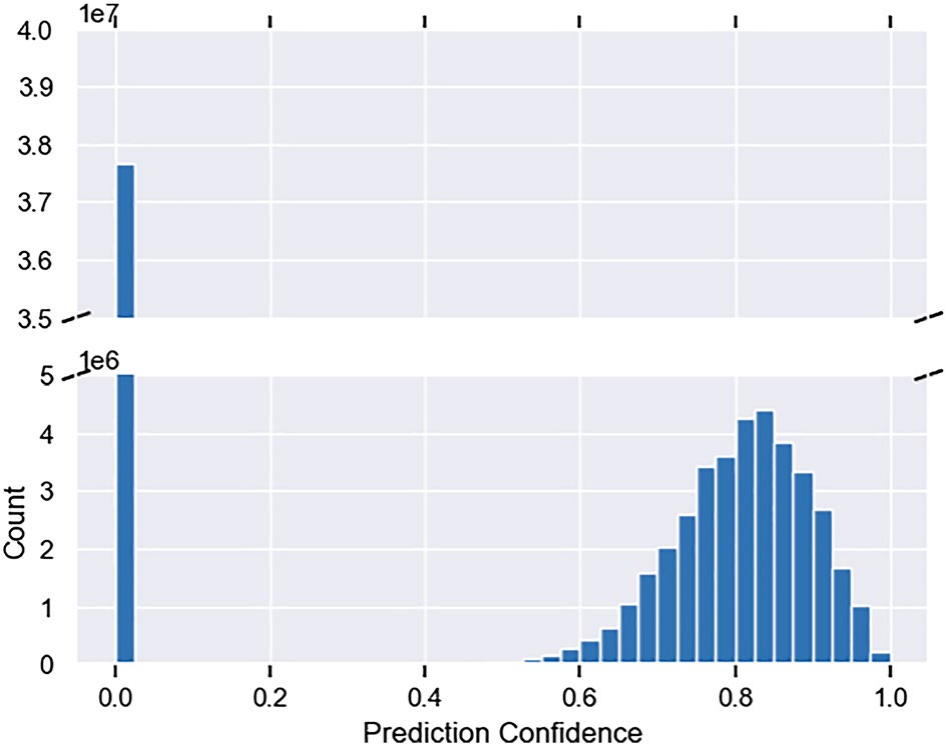
Distribution of miRNA target prediction confidences of the 3342 high-confidence SCN miRNA and all available SCN mRNA.

The application of miRdup to the high-confidence 3342 SCN pre-miRNA resulted in 6622 high-confidence mature SCN miRNA. TarPMir was applied to the 6622 high-confidence mature SCN miRNA and over 22,000 available SCN genes. Figure 7 displays the distribution of the resulting predictions after filtering to only include the highest confidence prediction for each miRNA: mRNA pair and those interactions that occur in the 3′ UTR region.

Although the CE-TarPMir predictor demonstrated improved target prediction performance on *C. elegans*, the same cannot be said for the intraspecies SCN target prediction. Using the CE-TarPMir target predictor resulted in a high number of “high-confidence” targets. It is expected that SCN intraspecies target prediction may benefit from an active learning approach where high-confidence targets are validated and added to the training data to iteratively improve classifier performance.

**Figure 8:**
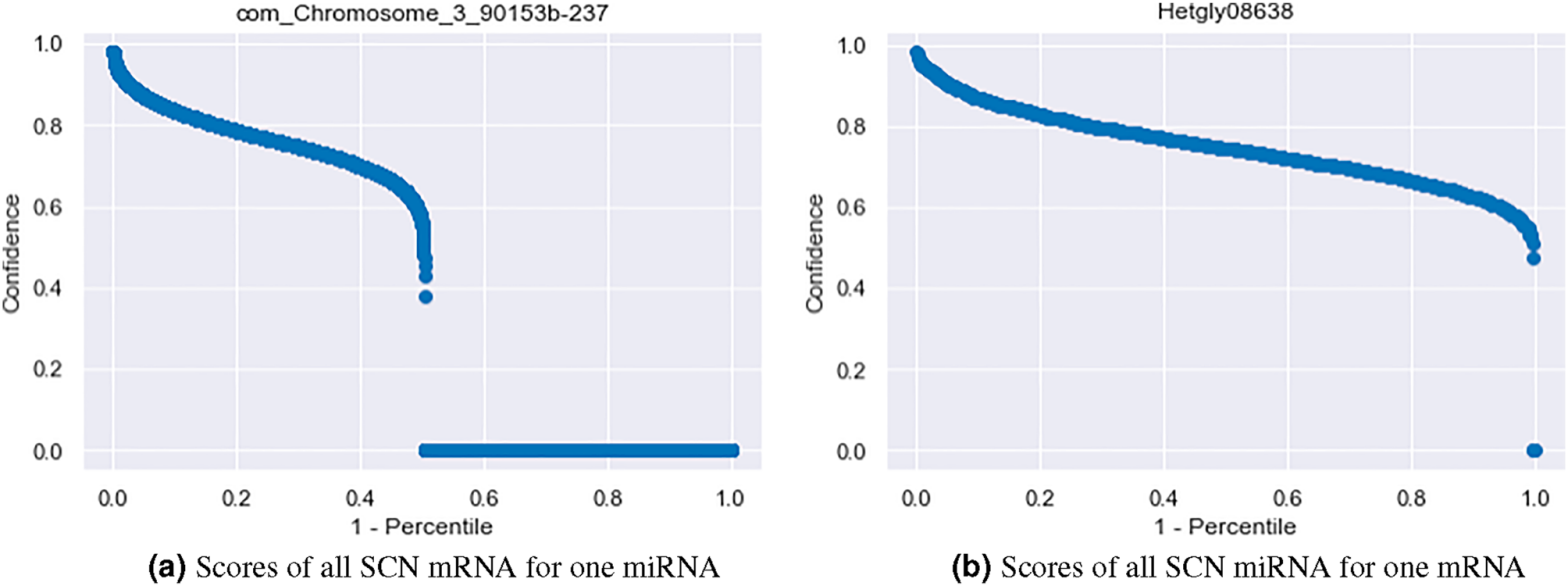
One-to-all curves for (**a**) an SCN candidate miRNA and (**b**) an SCN mRNA.

Qualitative reciprocal perspective was applied to the filtered SCN intraspecies interactions to produce high-confidence datasets resulting in the target prediction counts and percentage of total interactions seen in Table 7. Example one-to-all curves visualizing all the interaction prediction confidences for an SCN candidate miRNA and an SCN mRNA can be seen in Fig. 8. We leave it to the reader to determine the value of n to apply for the number of miRNA targets and mRNA interactions considered. One thing to consider is there exists a precision-recall trade-off, a higher n or m may increase the number of true targets recalled however it will reduce the precision of the dataset.

Among the predicted SCN mRNA targets using and n and m of 100, we found 970 sequences containing a secretion signal peptide with no transmembrane domain, a feature commonly associated with putative effectors. These mRNAs were targeted by an average of 6.5 miRNA and a maximum of 30^97^ . Several well-known SCN effectors were targeted by multiple miRNAs, for example Hetgly05453 (4D06, targeted by 21 miRNA), previously identified as pathogen-associated molecular pattern-triggered immunity (PTI) suppressors^97^ . PTI is the first layer of plant defense that the nematode needs to deactivate for successful invasion of its host. These miRNAs could therefore be a key element in the fine regulation of the expression of these genes.

**Table 7:** Qualitative reciprocal perspective applied to filtered SCN intra-species miRNA:mRNA targeting at various thresholds [n,m].

Pair falls within top-n predicted miRNA for mRNA							
n,m	1	4	8	10	25	50	100
Pair falls within top-m mRNA for the miRNA							
1	354 (0.002%)	677 (0.005%)	793 (0.005%)	819 (0.006%)	891 (0.006%)	908 (0.006%)	913 (0.006%)
4	999 (0.007%)	2243 (0.015%)	2845 (0.020%)	3019 (0.021%)	3477 (0.024%)	3595 (0.025%)	3638 (0.025%)
8	1456 (0.01%)	3729 (0.026%)	5031 (0.035%)	5414 (0.037%)	6666 (0.046%)	7086 (0.049%)	7242 (0.050%)
10	1615 (0.011%)	4307 (0.03%)	5972 (0.041%)	6468 (0.044%)	8162 (0.056%)	8782 (0.06%)	9042 (0.062%)
25	2249 (0.015%)	6953 (0.048%)	10,862 (0.075%)	12,270 (0.084%)	17,975 (0.123%)	20,828 (0.143%)	22,238 (0.153%)
50	2636 (0.018%)	9029 (0.062%)	15,413 (0.106%)	17,906 (0.123%)	29,908 (0.205%)	37826 (0.260%)	42,881 (0.294%)
100	2885 (0.020%)	10,750 (0.074%)	119,669 (0.135%)	23534 (0.162%)	44,865 (0.308%)	63,499 (0.436%)	78998 (0.542%)

#### SCN-Soy inter-species miRNA target prediction

**Figure 9:**
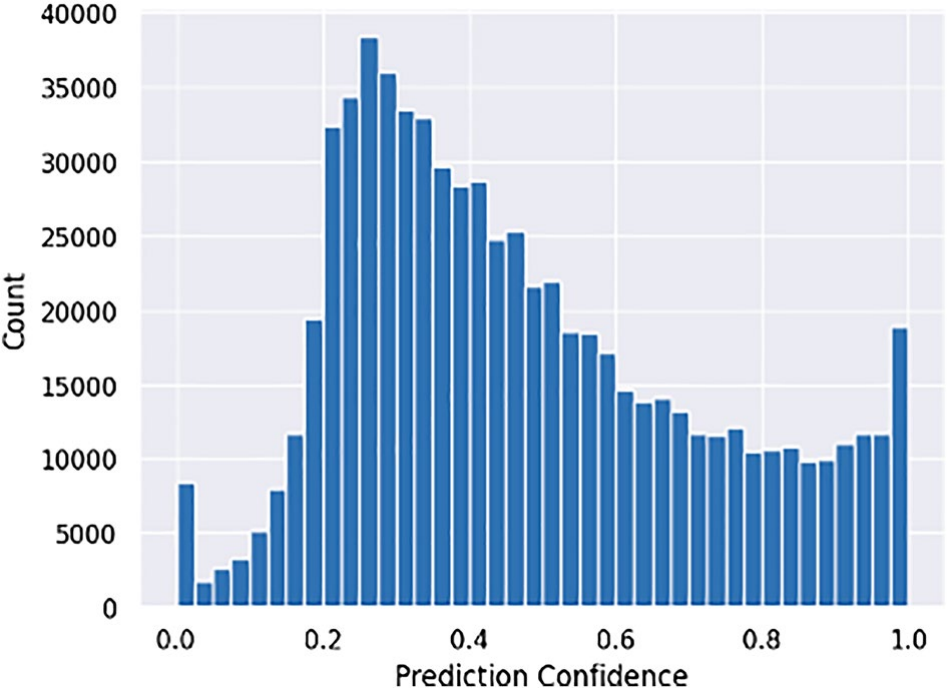
Distribution of miRNA target prediction confidences of the 3342 high-confidence SCN miRNA and 216 soybean mRNA.

P-TarPMir, the TarPMir classifier retrained with plant miRNA:mRNA targets, was applied to the  1 million pairs arising from the 6622 high-confidence mature SCN miRNA and the 216 soybean mRNA which could be involved in the defence against SCN. Figure 9 displays the distribution of the resulting predictions after filtering to only include the highest confidence prediction for each miRNA:mRNA pair.

**Figure 10:**
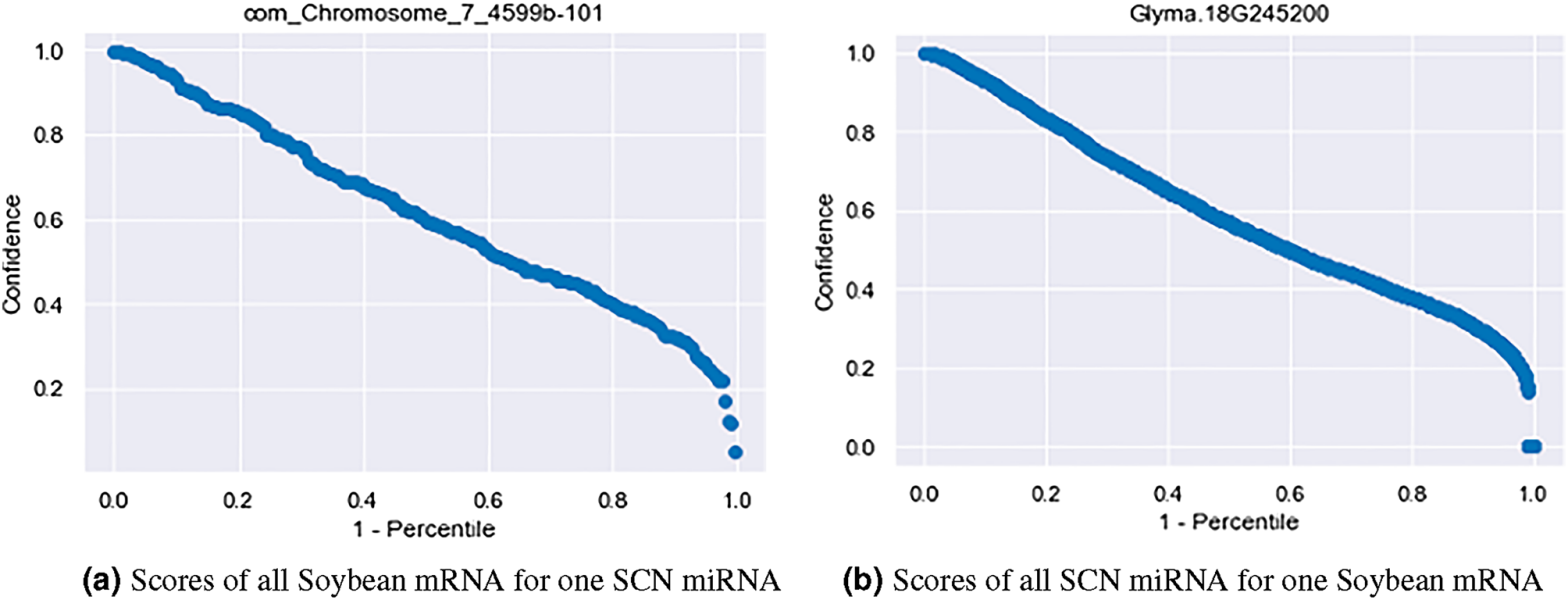
One-to-all curve for (**a**) SCN candidate miRNA and (**b**) Soybean mRNA.

Qualitative reciprocal perspective was also applied to the filtered SCN inter-species interactions with soybean resulting in the cumulative distribution seen in Table 8. One-to-all curves visualizing all the interaction confidences for an SCN candidate miRNA, and a soybean mRNA can be seen in Fig. 10. Similar to the SCN intra-species interactions, we leave it to the reader to decide the n,m thresholds to apply. The soybean genome has undergone two duplications, suggesting that four copies of a single mRNA may exist^98^ . Similar precision-recall considerations that apply to the intra-species interaction above also apply to the inter-species interactions here. Among the significant inter-species interactions, it is particularly interesting to note that the five miRNAs identified from intronic regions of known SCN effector genes were predicted to interact with 16 soybean genes that are potentially involved in SCN resistance^99^.

**Table 8:** Qualitative reciprocal perspective applied to filtered SCN inter-species miRNA:mRNA targeting at various thresholds [n,m].

Pair falls within top-n predicted miRNA for mRNA							
n,m	1	4	8	10	25	50	100
Pair falls within top-m mRNA for the miRNA							
1	79 (0.004%)	296 (0.013%)	582 (0.026%)	727 (0.032%)	1488 (0.066%)	2108 (0.094%)	2648 (0.118%)
4	145 (0.006%)	563 (0.025%)	1165 (0.052%)	1447 (0.064%)	3298 (0.146%)	5500 (0.244%)	8127 (0.361%)
8	166 (0.007%)	666(0.03%)	1365 (0.061%)	1708 (0.076%)	4108 (0.182%)	7419 (0.33%)	12,272 (0.545%)
10	171 (0.008%)	691 (0.031%)	1408 (0.063%)	1763 (0.078%)	4295 (0.191%)	7939 (0.353%)	13,603 (0.604%)
25	201 (0.009%)	800 (0.036%)	1581 (0.07%)	1970 (0.087%)	4855 (0.216%)	9457 (0.42%)	17,926 (0.796%)
50	208 (0.009%)	834 (0.037%)	1665 (0.074%)	2072 (0.092%)	5138 (0.228%)	10,118 (0.449%)	19,760 (0.878%)
100	209 (0.009%)	836 (0.037%)	1672 (0.074%)	2090 (0.093%)	5225 (0.232%)	10433 (0.463%)	20,746 (0.921%)

psRNATarget—a commonly used ab initio plant target predictor—was applied to the high-confidence SCN miRNA and the 216 soybean mRNA suspected to play a role in soybean pathogen defense. Table 9 shows the number of the high-confidence inter-species miRNA:mRNA interactions discovered by qualitative reciprocal perspective applied to the P-TarPMir predictions that were also found in the psRNATarget results with a relaxed expectation value.

**Table 9:** Interactions predicted by QRP applied to P-TarPMir and psRNATarget (Exp $\ge$ 3).

Pair falls within top-n predicted miRNA for mRNA							
n,m	1	4	8	10	25	50	100
Pair falls within top-m mRNA for the miRNA							
1	1	1	1	1	1	1	1
4	9	15	20	20	24	25	25
8	15	25	31	31	35	38	38
10	18	31	40	40	44	47	47
25	38	74	88	89	95	98	98
50	49	104	125	128	137	140	141
100	61	130	161	168	185	188	190

The predicted mRNA targets in soybean included a diverse array of gene functions. Certain functions such as transcription factors and response to oxidative stress were noticeably more prevalent. By employing a stringent criterion (top 8 by 8 reciprocal perspective) a total of 1365 pairs were identified, collectively targeting 246 distinct soybean genes. Among this set, 60 genes (24%) are associated with GO terms related to transcription factor activity. Notably, the GO term 0003700 (“DNA—binding transcription factor activity”) displayed significant enrichment with an adjusted p value of 6.85E-06. This group includes various WRKY and MYB genes that have demonstrated involvement in the interaction between SCN and soybean, as highlighted in the work of Hosseini and Matthews^100^. Furthermore, some of these genes exhibited reduced expression in different soybean lines when subjected to SCN infection, as observed in the study by Miraeiz et al.^101^, suggesting that they could be regulated by inter-species miRNA–mRNA interactions.

We acknowledge that the concept of cross-kingdom miRNA targeting is highly debated. Some researchers have suggested that the evidence of cross-kingdom targeting is most likely due to contamination of sequencing platforms rather than miRNA transfer in vivo (see^99^). Additionally, there exist mechanistic differences in the mode of action of plant and animal miRNAs. In this research, we explore the possibility of cross-kingdom miRNA– mRNA interactions utilizing a plant-trained classifier.

## Conclusion

This study has developed methods for miRNA discovery and target prediction for the Soybean Cyst Nematode, a destructive Soybean pathogen. In the face of no known miRNA within SCN, we developed SCN-specific miRNA discovery predictors, based on a species-specific dataset created using the SMIRP framework. This approach to creating species-specific miRNA predictors was validated for use on nematodes for the first time here, with precision and recall achieving 0.99 and 0.72, respectively, on the *C. elegans* model organism (CE-holdout experiment). A total of 3342 high-confidence candidate SCN miRNA are reported here.

MicroRNA target prediction was completed for two cases: intra-species within SCN and inter-species where SCN miRNA are hypothesized to interact with soybean mRNA. The TarPMir miRNA target prediction approach is tailored through fine-tuning for both the plant and nematode cases. To increase the specificity of predicted miRNA:mRNA interactions, a qualitative reciprocal perspective approach is introduced. Focusing on the genes potentially involved in SCN pathogenicity, we found that they are predicted to interact with multiple miRNAs (up to 30) which could regulate their expression. Moreover, some of these genes appear to host miRNA precursors in their intronic regions that, in turn, target resistance genes in soybean or other effector genes in the nematode, suggesting a complex regulatory cascade.

In summary, we have developed custom methods for miRNA discovery in an important Soybean pathogen and for miRNA target prediction within SCN and between SCN and soybean. These methods, along with the high-confidence predictions, are expected to be of great interest to those studying SCN, soybean and other plant pathogens that may be mediated by miRNA post-transcriptional gene regulation.

### Supplementary Information

Supplementary Information 1.

Supplementary Information 2.

Supplementary Information 3.

Supplementary Information 4.

Supplementary Information 5.

Supplementary Information 6.

## Data Availability

All predicted miRNA and mRNA targets are available in a Borealis Dataverse Repository at https://borealisdata.ca/dataset.xhtml?persistentId=doi:10.5683/SP3/30DEXA. Code for miRNA discovery and target prediction is available at https://github.com/GreenCUBIC/SMIRP_SCN.
