# Supplementary figures and images for: Species-specific microRNA discovery and target prediction in the soybean cyst nematode
Source: Sci Rep. 2023 Oct 17;13:17657. doi: 10.1038/s41598-023-44469-w (PMC10582106; doi:10.1038/s41598-023-44469-w)

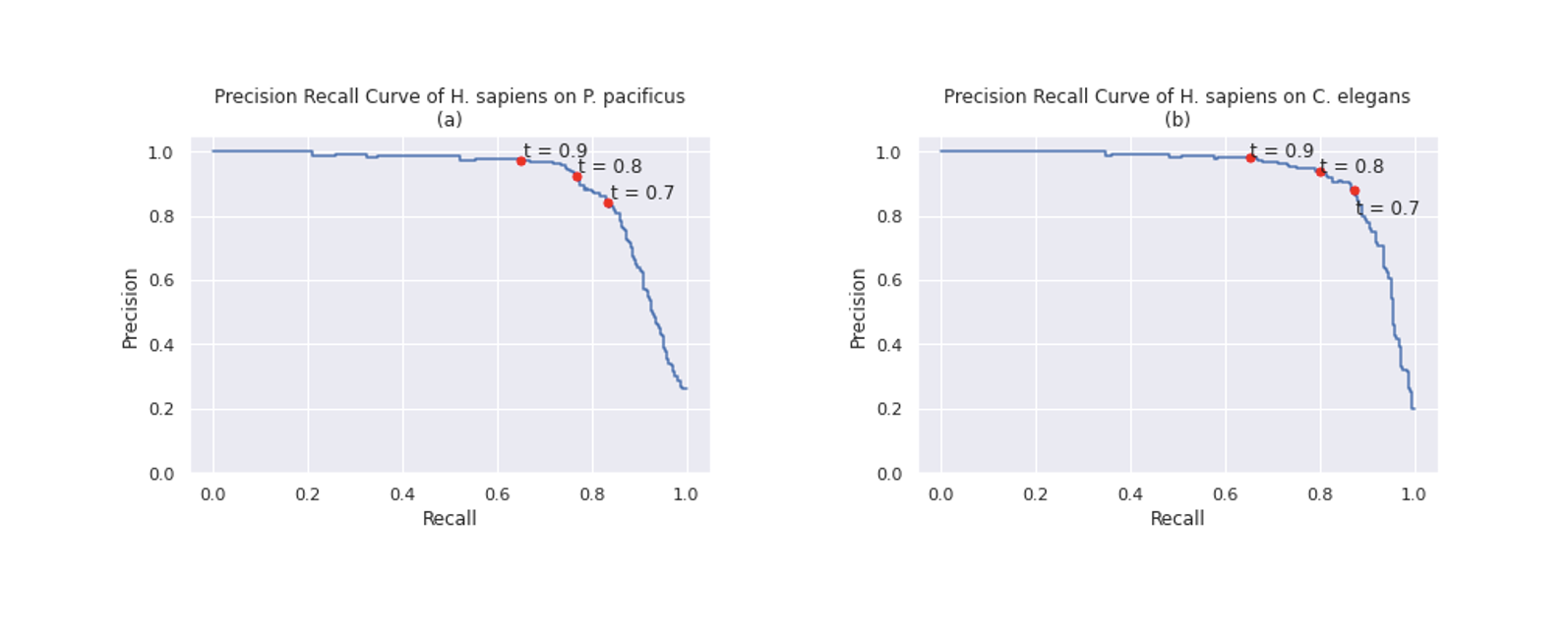

Supplement: Supplementary file 1 — Supplementary Information 1. [file 41598_2023_44469_MOESM1_ESM.png]

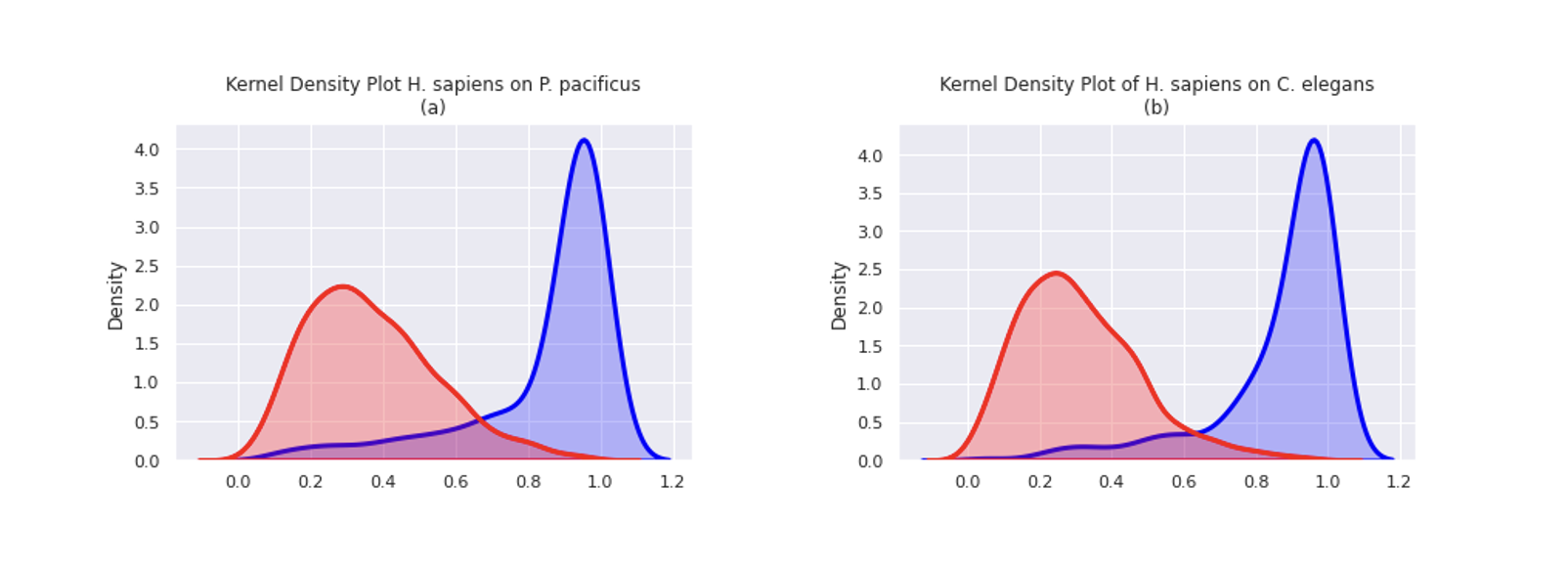

Supplement: Supplementary file 2 — Supplementary Information 2. [file 41598_2023_44469_MOESM2_ESM.png]
