# Supplementary material for: Species-specific microRNA discovery and target prediction in the soybean cyst nematode
Source: Sci Rep. 2023 Oct 17;13:17657. doi: 10.1038/s41598-023-44469-w (PMC10582106; doi:10.1038/s41598-023-44469-w)
Supplement: Supplementary file 3 — Supplementary Information 3. [file 41598_2023_44469_MOESM3_ESM.docx]

**Gene lists for miRNA project**

The list of soybean genes were determined based on literature curation as broken down in supplementary file # as well as from Soybase’s GWAS QTL page under SCN 1 - SCN 6 **(**[**https://soybase.org/GWAS/list.php#S**](https://soybase.org/GWAS/list.php#S)**).** Gene names as in version Wm82.a2.v1.

**Soybean control genes Rhg1 and Rhg4 genes**

Glyma.18g022400 (rhg1)

Glyma.18g022500 (rhg1)

Glyma.18g022600 (rhg1)

Glyma.08g109000 (rhg4)

Glyma.08g187800 (rhg4)

Glyma.08g108900 (rhg4)

**Soybean genes:**

**List of genes in SoyBase’s GWAS QTL page “SCN 1- SCN 6” (**[**https://soybase.org/GWAS/list.php#S**](https://soybase.org/GWAS/list.php#S)**)**

| Glyma.07g195300 |
| --- |
| Glyma.07g199900 |
| Glyma.07g191200 |
| Glyma.07g191500 |
| Glyma.09g279100 |
| Glyma.10g173000 |
| Glyma.10g173100 |
| Glyma.10g081700 |
| Glyma.13g117600 |
| Glyma.13g172600 |
| Glyma.15g051600 |
| Glyma.18g064100 |
| Glyma.18g210500 |
| Glyma.18g017800 |
| Glyma.18g252300 |
| Glyma.18g252400 |
| Glyma.20g193500 |
| Glyma.03g170000 |
| Glyma.04g063600 |
| Glyma.04g018500 |
| Glyma.04g211300 |
| Glyma.04g211000 |
| Glyma.07g194200 |
| Glyma.07g194800 |
| Glyma.07g195000 |
| Glyma.07g195700 |
| Glyma.07g195900 |
| Glyma.07g191000 |
| Glyma.07g193900 |
| Glyma.07g195500 |
| Glyma.08g100100 |
| Glyma.08g100800 |
| Glyma.08g202300 |
| Glyma.10g172800 |
| Glyma.10g173300 |
| Glyma.10g211400 |
| Glyma.11g227500 |
| Glyma.13g266600 |
| Glyma.14g048600 |
| Glyma.14g048500 |
| Glyma.14g103100 |
| Glyma.15g252000 |
| Glyma.18g022300 |
| Glyma.18g106800 |
| Glyma.18g107000 |
| Glyma.18g196200 |
| Glyma.18g210300 |
| Glyma.18g011600 |
| Glyma.18g028600 |
| Glyma.18g040500 |
| Glyma.20g024100 |
| Glyma.07g171000 |
| Glyma.04g200900 |
| Glyma.04g211400 |
| Glyma.06g147000 |
| Glyma.07g194400 |
| Glyma.07g195100 |
| Glyma.07g199000 |
| Glyma.07g199500 |
| Glyma.07g199700 |
| Glyma.07g195400 |
| Glyma.07g196000 |
| Glyma.07g196800 |
| Glyma.07g193300 |
| Glyma.08g097300 |
| Glyma.08g099700 |
| Glyma.08g200100 |
| Glyma.08g200200 |
| Glyma.08g200800 |
| Glyma.08g201100 |
| Glyma.09g278900 |
| Glyma.10g172700 |
| Glyma.10g172900 |
| Glyma.10g173400 |
| Glyma.13g157900 |
| Glyma.13g172700 |
| Glyma.14g047900 |
| Glyma.14g049500 |
| Glyma.14g051600 |
| Glyma.14g219500 |
| Glyma.18g063400 |
| Glyma.18g063500 |
| Glyma.18g077900 |
| Glyma.18g078000 |
| Glyma.18g107100 |
| Glyma.18g193200 |
| Glyma.18g193300 |
| Glyma.18g193400 |
| Glyma.18g193800 |
| Glyma.18g203500 |
| Glyma.18g010000 |
| Glyma.18g023700 |
| Glyma.18g252200 |
| Glyma.18g278200 |
| Glyma.19g094100 |
| Glyma.19g119300 |
| Glyma.19g120200 |
| Glyma.19g012900 |

**qSCN18 fine mapping paper by Usovsky et al. 2021 (**[**https://link.springer.com/content/pdf/10.1007/s00122-020-03718-6.pdf**](https://link.springer.com/content/pdf/10.1007/s00122-020-03718-6.pdf)**)**

| Glyma.18g243000 |
| --- |
| Glyma.18g243100 |
| Glyma.18g243200 |
| Glyma.18g243300 |
| Glyma.18g243400 |
| Glyma.18g243500 |
| Glyma.18g243600 |
| Glyma.18g243700 |
| Glyma.18g243800 |
| Glyma.18g243900 |
| Glyma.18g244000 |
| Glyma.18g244100 |
| Glyma.18g244200 |
| Glyma.18g244300 |
| Glyma.18g244400 |
| Glyma.18g244500 |
| Glyma.18g244600 |
| Glyma.18g244700 |
| Glyma.18g244800 |
| Glyma.18g244900 |
| Glyma.18g245000 |
| Glyma.18g245100 |
| Glyma.18g245200 |

**GWAS paper by Tran et al. 2019 (**[**https://www.frontiersin.org/articles/10.3389/fpls.2019.00401/full**](https://www.frontiersin.org/articles/10.3389/fpls.2019.00401/full)**)**

Glyma.07g194200

Glyma.07g194400

Glyma.07g194800

Glyma.07g195000

Glyma.07g195100

Glyma.07g195300

Glyma.07g195400

Glyma.07g195500

Glyma.07g195700

Glyma.07g195900

Glyma.07g196000

Glyma.07g196500

Glyma.07g199000

Glyma.07g200100

Glyma.07g199500

Glyma.07g199700

Glyma.07g199900

Glyma.10g172900

Glyma.10g173000

Glyma.10g173100

Glyma.10g173300

Glyma.10g173400

**RNA-seq paper by Jiang et al. 2020 (**[**https://complete.bioone.org/journals/Crop-and-Pasture-Science/volume-71/issue-6/CP20060/RNA-Seq-based-identification-of-potential-resistance-mechanism-against-the/10.1071/CP20060.full**](https://complete.bioone.org/journals/Crop-and-Pasture-Science/volume-71/issue-6/CP20060/RNA-Seq-based-identification-of-potential-resistance-mechanism-against-the/10.1071/CP20060.full)**)**

| Glyma.10G165800 |
| --- |
| Glyma.03G112000 |
| Glyma.19G178200 |
| Glyma.08G227700 |
| Glyma. 06G092000 |
| Glyma.05G165800 |
| Glyma. 06G003800 |
| Glyma.03G141200 |
| Glyma.15G135600 |
| Glyma. 10G067000 |
| Glyma.06G142000 |
| Glyma.09G274000 |
| Glyma.18G213200 |

**Transcriptomic and metabolomic paper by Kang et al. 2018 (**[**https://bmcplantbiol.biomedcentral.com/track/pdf/10.1186/s12870-018-1302-9.pdf**](https://bmcplantbiol.biomedcentral.com/track/pdf/10.1186/s12870-018-1302-9.pdf)**)**

| GLYMA.20G114200 |
| --- |
| GLYMA.10G209800 |
| GLYMA.10G058200 |
| GLYMA.13G145000 |
| GLYMA.01G232400 |
| GLYMA.11G010500 |
| GLYMA.09G022300 |
| GLYMA.13G307000 |
| GLYMA.12G195500 |
| GLYMA.10G222400 |
| GLYMA.12G195600 |
| GLYMA.09G284700 |
| GLYMA.09G156700 |
| GLYMA.06G302700 |
| GLYMA.01G130500 |
| GLYMA.03G038700 |
| GLYMA.10G262400 |
| GLYMA.10G172700 |
| GLYMA.11G045600 |
| GLYMA.11G021500 |
| GLYMA.08G018000 |
| GLYMA.05G37410 |
| GLYMA.07G128000 |
| GLYMA.15G112700 |
| GLYMA.02G268200 |
| GLYMA.14G049500 |
| GLYMA.05G151100 |
| GLYMA.18G261600 |
| GLYMA.17G184900 |
| GLYMA.19G114500 |
| GLYMA.08G282800 |
| GLYMA.08G024700 |
| GLYMA.18G232000 |
| GLYMA.03G009800 |
| GLYMA.05G012300 |
| GLYMA.18G156100 |

**Early transcriptional response paper by Miraeiz et al. 2020 (**[**https://link.springer.com/content/pdf/10.1007/s00122-019-03442-w.pdf**](https://link.springer.com/content/pdf/10.1007/s00122-019-03442-w.pdf)**)**

Glyma.01G128100

Glyma.03G042700

Glyma.05G215900

Glyma.06G303100

Glyma.19G257400

Glyma.11G194100

Glyma.12G218200

Glyma.13G282100

Glyma.13G302400

Glyma.02G005600

Glyma.10G006600

Glyma.10G180800

Glyma.16G023000

Glyma.04G04490

Glyma.06G045400

Glyma.04G044900

Glyma.17G0064700
